# From Thriving Developers to Stagnant Self-Doubters: An Identity-Centered Approach to Exploring the Relationship Between Digitalization and Professional Development

**DOI:** 10.1007/s12186-022-09288-6

**Published:** 2022-03-25

**Authors:** Anna Wallin, Petri Nokelainen, Mari Kira

**Affiliations:** 1 Faculty of Education and Culture, Tampere University, 33014 Tampere, Finland; 2 Department of Psychology, University of Michigan, MI 48109 Ann Arbor, USA

**Keywords:** Professional identity, Professional development, Identity work, Job crafting, Digitalization, The method of empathy-based stories

## Abstract

This article reports a study illustrating the relationship between digitalization and professional development from an identity-centered perspective. Drawing on a unique data set of 101 empathy-based stories from 81 Finnish government workers, the findings show how workers might experience and respond to work-identity alignments and misalignments in a digitalized working life and how this might influence their professional development. We identify four typifications—the thriving developer, the loyal transformer, the stagnant self-doubter, and the career crafter— and illustrate how digitalization can either support or hinder professional development by inducing work-identity (mis)alignments and how workers may respond to these in different ways by engaging in identity work and job crafting. In particular, our findings emphasize the role professional identity and agency play in professional development and highlight the importance of recognizing how digitalization of work can threaten or support workers’ professional identities to build a supportive working environment where the workers feel like they are valued and able to develop in a meaningful way.

## Introduction

In today’s working life, workers are increasingly challenged to continuously develop and learn as digitalization transforms their work tasks, practices, and methods of communication in various ways (e.g., Parker & Grote, 2020). Digitalization is a broad concept, referring to how digital technologies (such as information and communication technology (ICT), artificial intelligence (AI) and robotics) can lead to fundamental changes, altering for instance existing business processes such as communication (e.g., Verhoef et al., 2021). In this article, we approach digitalization from the perspective of knowledge workers, for whom digitalization of work is especially visible in the growing use of ICTs, resulting in changes in information and knowledge sharing, management, and communication (e.g., Vuori et al., 2019). With the use of ICTs, accessing, sharing, gathering, and analyzing information becomes easier, and employees will find new ways to collaborate and work regardless of time and place (e.g., Fischer et al., 2018). Indeed, in knowledge work, digitalization is often linked to flexible work designs, referred to as smart work (e.g., Raguseo et al., 2016) and new ways of working (de Leede, 2016), which can be defined as “practices in which employees are able to work independent of time, place and organization, supported by a flexible work environment which is facilitated by information technologies” (de Leede, 2016, p. xiii).

In addition to changes in work practices, digitalization of work has prompted much discussion regarding how automation in the form of AI and robotics potentially alters job skill demands and the labour market as machines substitute humans (e.g., Autor, 2015; Brynjolfsson et al., 2018; Frey & Osborne, 2017). Estimations have ranged from more extreme views demonstrating how automation may lead to massive unemployment and job destruction (e.g., Frey & Osborne, 2017) to more cautious ones, arguing that despite that most jobs have some tasks that can be automatable, few jobs are fully automatable (Brynjolfsson et al., 2018). Especially jobs involving routine work tasks and a lower level of complexity has been suggested to be more likely automated compared to tasks requiring highly cognitive skills (Autor, 2015; Frey & Osborne, 2017; Goller & Harteis, 2017). However, with the combination of big data and AI, higher-skill domains are not completely safe from automation, meaning that more complex knowledge work and management could potentially be replaced by AI (e.g., Parker & Grote, 2020). Despite the controversies between these views, they all share the view that digitalization of work fundamentally alters how we think about work and requires the workers to deal with new work tasks and practices where people and digital technologies work together (e.g., Parker & Grote, 2020). Thus, for knowledge workers, digitalization of work may contribute to a change in their work tasks and to a major shift of view on how, where, and when work is performed, requiring workers to work in new, distributed and continuously changing work environments that are technologically mediated.

At the same time as digitalization of work changes the ways of working (what workers do), it also challenges the views that workers hold of themselves as workers (who they are). In other words, digitalization challenges their professional identities (see, e.g., Nach, 2015; Walsham, 1998). When workers are experiencing transformations in their work (such as described above), workers are required to constantly assess whether their professional identities match their transformed work context (Kira & Balkin, 2014). These experienced work-identity (mis)alignments and their possible relation to workers’ professional development is in the main focus of this article.

Although previous studies exploring the relationship between digitalization and professional learning and development have provided valuable insights into how digitalization shapes workers’ competence requirements and workplaces as learning environments (e.g., Fischer et al., 2018; Harteis, 2018; Vallo Hult & Byström, 2021; Wallin et al., 2020), the role of workers’ identities in the interplay between digitalization and professional development is still poorly understood. Given that the current view of professional development holds that a shift is needed from a “training model” toward a view that emphasizes workers’ self-directedness, identities, and agency in learning and development (Boud & Hager, 2012; Eteläpelto, 2017; Goller & Harteis, 2017; Vähäsantanen & Hämäläinen, 2019; Webster-Wright, 2009), exploring the relationship between digitalization and professional identity becomes important in building an understanding of how digitalization may influence workers’ professional development.

Therefore, in this article professional development is understood from a broad, life-long learning perspective, emphasizing the continuous and practice-based nature of professional development, i.e., how workers continuously learn across the various stages of their career. The roles that professional agency and identity play in professional development become highlighted, as the focus expands from professional development programs and a ‘transfer’ training metaphor toward a view emphasizing a constant process of learning and ‘becoming’ in professional development (Boud & Hager, 2012; Havnes & Smeby, 2014; Webster-Wright, 2009). We adopt an identity-centered approach, in which professional development does not only comprise of acquiring knowledge, developing competencies, or updating skills but also involves questions related to how workers define themselves as professionals, such as “Who am I,” “What do I do,” and “Who do I want to become?” (Akkerman & Meijer, 2011; Eteläpelto et al., 2014; Vähäsantanen & Billett, 2008; Vähäsantanen & Hämäläinen, 2019). Workers strive to maintain a positive identity and a situation where their identity aligns with their work context and, to achieve this, they continuously craft their jobs to better fit their needs and interests, and they engage in identity work to explore the meaning of their professional identity (Kira & Balkin, 2014). In line with the subject-centered socio-cultural approach (e.g., Eteläpelto et al., 2014), we understand workers as active agents, who exert their professional agency by influencing, making choices, and taking stances in ways that affect their work and their professional identities (p. 659), i.e., by engaging in identity work and job crafting. Thus, in this article professional development is understood as a continuous process in which workers maintain and develop their identities and ways of working by engaging in identity work and job crafting under the socio-cultural and material conditions of the workplace.

The aim of this study is to explore workers’ various possible experiences and responses to work-identity alignments and misalignments in a digitalized working life and how these experiences and responses might support or hinder their professional development. To empirically investigate the topic, we collected qualitative data with a novel data-gathering method called the method of empathy-based stories (MEBS; e.g., Wallin et al., 2019). In accordance with narrative research (e.g., Bruner, 2004), the MEBS builds on the idea that storytelling can enhance people’s capabilities to express and share perceptions, meanings, and tacit understandings. Thus, by exploring empathy-based stories (*N* = 101) written by Finnish government workers, we aim to provide insights into individual sensemaking and to explore shared perceptions and beliefs that the participants associate with the phenomenon. Rather than constructing a detailed and comprehensive model or testing hypotheses, the objective of this research is to illustrate possible and compelling scenarios and connections and thus widen the understanding of and guide future research on this emerging topic.

## Theoretical Perspectives

### Work-Identity (mis)alignments Amid Digitalization of Work

In a broad sense, identity refers to “the meanings that individuals attach reflexively to themselves” (Brown, 2015, p. 23) and implies “what is appropriate, natural and valued for a specific subject” (Kärreman & Alvesson, 2001, p. 64). Thus, professional identity relates to how individuals define themselves as professionals, and comprise professionals’ subjective goals, experiences, interests, values, knowledge, competencies, commitments, and future career prospects (e.g., Eteläpelto et al., 2014; Fitzgerald, 2020; Vähäsantanen & Hämäläinen, 2019). Professional identities are often studied in the context of specific professions (e.g., teachers, lawyers, and doctors). As such, the definition of professional identity often includes the characteristics of a specific profession. In this article, we use the term professional identity at a general level and in a broad sense (not tied to a specific profession and adopting a loose definition of the term “professional”; e.g., Havnes & Smeby, 2014), referring to a “professional employee’s identities in relation to their work, as opposed to hobby-like activities” (Eteläpelto et al., 2014, p. 649). As such, our understanding of professional identity in this article is closely related to the definition of work identity, which refers to “a work-based self-concept, constituted of a combination of organizational, occupational, and other identities, that shapes the roles individuals adopt and the corresponding ways they behave when performing their work in the context of their jobs and/or careers” (Walsh & Gordon, 2008, pp. 47–48).

While professional identity is based on individual subjectivities, a dialogical relationship exists between identity and work environment. Workers constantly confront different contradictions, frustrations, and (role) expectations in their work environment, and identity construction can be seen as a “struggle” (Alvesson, 2010; Arvaja, 2016) occurring through a continuous dialogical process of positioning oneself between the self and the social context (Akkerman & Meijer, 2011; Arvaja, 2016). Thus, professional identities are agentically and socially reconstructed as both personal and contextual factors shape how professionals negotiate their identities and how they view themselves as professionals (e.g., Kira & Balkin, 2014; Vähäsantanen & Billett, 2008).

Acknowledging the dialogical relationship between professional identity and the work environment, we draw on research on work-identity interactions. In particular, we adapt the model by Kira and Balkin (2014), which outlines how encounters between work and identity can induce different experiences, reactions, and outcomes. This model builds on the idea that workers tacitly and explicitly assess whether their work aligns or misaligns with their (professional) identity and may consequently experience a match or mismatch between their professional identity and work context (Kira & Balkin, 2014). In an ideal situation, a person’s work is aligned with the person’s professional identity. A harmonious relationship between work and professional identity is established as the person can manifest their professional interests and feels competent in their work (Vähäsantanen & Hämäläinen, 2019). Thus, when changes at work enable a worker to experience a harmonious work-identity alignment, for instance, by providing the worker with opportunities to realize their professional interests, the worker is positively disposed to changes and adopts an approving position toward changes (e.g., Vähäsantanen & Eteläpelto, 2011). Consequently, the person experiences thriving, meaningfulness, and satisfaction, and a feeling of being “in the right job” (Arvaja, 2016; Kira & Balkin, 2014; Pratt et al., 2006).

Regarding digitalization, earlier studies have found that the use of information communication technologies (ICTs) may be identity enhancing because they can provide opportunities for reskilling, knowledge development, and professional growth (Alvarez, 2008; Lamb & Davidson, 2005; Mishra et al., 2012). In these situations, technologies are seen as aligning with workers’ professional identities; workers see them as a means to fulfill their professional interests and as valuable to their professional identities (Nach, 2015; Stein et al., 2013). For instance, workers may view themselves as helpful mediators, adventurous and empowered creators, active agents, gatekeepers, or wise teachers in relation to information technologies (ITs), shaping their IT use and professional identities (Stein et al., 2013). Likewise, in a situation where the management demands the use of new technologies, workers may engage with technologies because they see themselves as “dedicated social conscious professionals, motivated by an intense work ethic and commitment to their job” (Leclercq-Vandelannoitte, 2014, p. 60). Thus, technologies can reinforce workers’ (professional) identities in multiple ways; by providing workers opportunities to, for instance, be a better worker by cutting down errors (e.g., Mishra et al., 2012), or by giving them a sense of importance and recognition (e.g., Leclercq-Vandelannoitte, 2014). Consequently, according to Stein et al. (2013),

IT becomes important for professional identity performance when particular *signs and functions* presented by the IT *align* with the professional’s *personal preferences* (what kind of work do they *want* to do, how they *want* to be known) and the *normative expectations* of the professional. (p. 179, emphasis in original)

Studies exploring work-identity encounters have focused more on the misalignments between work and identities than on their alignments (e.g., Kira & Balkin, 2014; Pratt et al., 2006; Sveningsson & Alvesson, 2003). Here, contextual changes in work may result in a work-identity mismatch as the workers’ view of “who they are” as professionals no longer matches the work that they do (Arvaja, 2016; Pratt et al., 2006). Workers may, for instance, lack the necessary competencies for their current job or experience a lack of opportunities to use their full potential (Arvaja, 2016; Kira & Balkin, 2014; Pratt et al., 2006; Vähäsantanen & Hämäläinen, 2019). For example, ICTs may threaten and challenge workers’ professional identities by deskilling professionals and making some areas of expertise obsolete (Alvarez, 2008; Lamb & Davidson, 2005; Nach, 2015), threatening their status positions and professional autonomy (Mishra et al., 2012). When workers experience a misalignment, a strained relationship between work and professional identity is established (Vähäsantanen & Hämäläinen, 2019). Consequently, workers may feel useless, inadequate, devalued, anxious, and frustrated (Kira & Balkin, 2014; Pratt et al., 2006; Vähäsantanen & Hämäläinen, 2019).

### Responses to Work-Identity (mis)Alignments

*Maintaining and transforming identity work.* In general, individuals strive for self-continuity and self-coherence (e.g., Burke & Stets, 2009) by engaging in identity work*,* which refers to “being engaged in forming, repairing, maintaining, strengthening or revising the constructions that are productive of a sense of coherence and distinctiveness” (Sveningsson & Alvesson, 2003, p. 1165). Several studies have demonstrated how identity work is an integral part of professional development (e.g., Eteläpelto et al., 2014; Pratt et al., 2006; Vähäsantanen et al., 2017), for instance, when workers engage in identity work, they learn more about themselves as professionals, how to use their strengths at work, how take new career directions and how to find meaning in their work (e.g., Kira & Balkin, 2014; Vähäsantanen, 2015; Vähäsantanen et al., 2017).

When workers experience their work and identities as aligning, they are likely to engage in maintaining identity work, which ensures self-continuity and aims at strengthening their present and future possibilities for meaningful work (Kira & Balkin, 2014). Maintaining or retaining an identity is generally about “general upkeep – sustaining, bolstering, or continuing to validate an identity” (Lepisto et al., 2015). For instance, a recent research illustrated how severely disabled soldiers strived to maintain their soldier identity through the continuity of their goals, values, and jobs when facing involuntary career transitions (Kulkarni, 2020). In a misalignment situation, workers may engage in transformative identity work to shape their identities to correspond with social cues (Kira & Balkin, 2014; Pratt et al., 2006). For instance, Nach (2015) found that workers engage in transformative identity work when their identity is challenged by the use of ITs. When workers feel like they have some control over the possibilities to adjust to this identity-threatening situation, they may end up reframing their identities successfully with respect to the IT capabilities and requirements. However, such a redefined identity is not always achieved although workers feel they have some control over the situation and engage in identity-adjusting mechanisms (e.g., efforts to learn new digital skills). In such a situation, workers form ambivalent identities, which are characterized by conflicting thoughts, feelings, and actions.

*Job crafting.* Transformative identity work is both cognitively and emotionally taxing as it requires workers to critically question their self-definitions (Kira & Balkin, 2014). Therefore, when experiencing a misalignment, workers often avoid transforming their identities and instead strive first to influence the conditions of their work (Kira & Balkin, 2014; Vähäsantanen & Billett, 2008). Thus, workers engage in job crafting—transforming their work context to align it better with their identities. Likewise, in a situation where workers experience work-identity alignments, they may engage in job crafting by, for instance, strengthening the aspects of the work situation that they experience as meaningful (Kira & Balkin, 2014). In several studies, job crafting has been connected to professional learning and development (e.g., Fuller & Unwin, 2017; Goller, 2017; Goller & Billett, 2014; Lazazzara et al., 2020). When engaging in job crafting, workers exert their professional agency, for instance, in negotiating the contents and conditions of their work and thus, learn more about their work and also about themselves (e.g., Kira & Balkin, 2014), thereby facilitating their professional learning and development.

Job crafting can take three forms: task, relational, and cognitive crafting (Wrzesniewski & Dutton, 2001). Task crafting refers to job-related changes that result in a different number, scope, or type of job tasks. Relational crafting involves changes in the quality and/or quantity of interactions with others at work, and cognitive crafting^1^ refers to reframing how one sees the job. Later, other job crafting conceptualizations have also been presented (e.g., Bruning & Campion, 2018; Tims & Bakker, 2010). Most recently, Lazazzara et al. (2020) integrated these by forming a process model that connects the motives, context, personal factors, and consequences of job crafting based on a distinction between approach and avoidance behaviors. They stated that “*approach crafting* is directed toward solving problems, improving the work situation, and accepting and interpreting stressors in a positive way, whereas *avoidance crafting* seeks to reduce or eliminate aspects of the job” (Lazazzara et al., 2020, p. 4). Approach task crafting includes strategies such as taking on extra tasks and responsibilities, whereas avoidance task crafting refers to reducing the number of tasks and responsibilities. Similarly, approach relational crafting can refer to creating additional relationships, whereas avoidance relational crafting can refer to reducing relationships and interaction. Moreover, approach cognitive crafting can refer to, for instance, emphasizing the positive qualities of work, whereas avoidance cognitive crafting can refer to acceptance of negative things or withdrawal crafting.

According to Lazazzara et al. (2020), workers engage in approach or avoidance crafting depending on whether their work context elicits proactive or reactive motives. Proactive motives refer to “employees wanting to initiate job crafting to reach desirable goals, while reactive motives are related to the need to cope with adversity” (Lazazzara et al., 2020, p. 10). Examples of desirable goals include improving one’s self-image, developing knowledge, and realizing career aspirations. Adversities, on the other hand, consist of hindrances to the experience of authenticity and various negative job characteristics, such as a lack of autonomy and high workload. Thus, whether workers engage in approach or avoidance crafting depends not only on their motives but also on the work context. Nevertheless, certain supportive factors in the work context (e.g., high social support and supportive job design) can encourage workers to engage in approach crafting even in situations where adversarial work-identity misalignments exist. However, when workers face a constraining context (e.g., low social support and high pressure to behave in a prescribed manner), they are more likely to engage in avoidance crafting or even stop any crafting attempts (Lazazzara et al., 2020).

Thus, a situation where workers experience both work-identity misalignments and a constraining context raises cynicism as workers cannot resolve the misalignments and “only recognize how they are expected to be something that they are not and do not want to be or cannot be as employees” (Kira & Balkin, 2014, p. 139). This might lead workers to adapt to the situation, distance themselves from their work, or withdraw themselves from their current organization or even their profession (Vähäsantanen & Billett, 2008; Vähäsantanen & Eteläpelto, 2011; Ylijoki & Ursin, 2013). In other words, workers engage in a form of avoidance cognitive crafting called withdrawal crafting, which means distancing oneself either mentally or physically from a person, situation, event, or environment (Bruning & Campion, 2018; Lazazzara et al., 2020). When ITs threaten workers’ professional identities, they may form “anti-identities” by completely disassociating themselves “from the meanings brought by IT” (Nach, 2015, p. 715). Thus, anti-identities are a result of resistance and allow workers to experience some control over the situation. Workers may also feel like they have no control over the situation and lack resources to cope with the identity-threatening situation. In this case, workers are overwhelmed by negative emotions, such as frustration and unworthiness, and feel that there is nothing they can do to change the situation (Nach, 2015).

## Research Questions

**Fig. 1:**
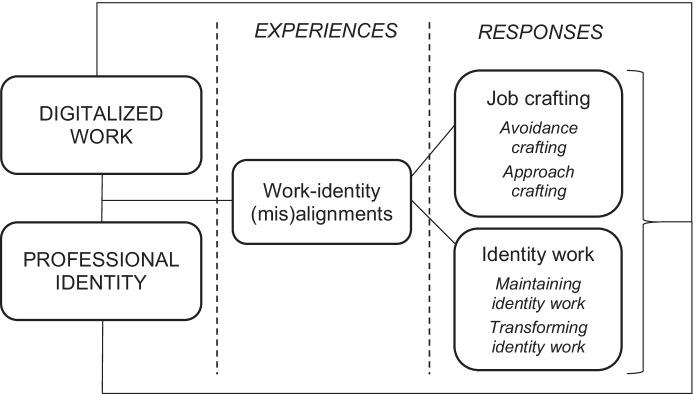
Professional development process

This study aims to contribute to research on professional development and work-identity interactions in the context of digitalized work by illustrating how digitalization might shape interactions between work and professional identity and consequently influence workers’ professional development. As discussed earlier, in this article professional development is understood as a continuous process in which professional development happens throughout workers’ careers as encounters between (digitalized) work and professional identity induce different experiences of work-identity (mis)alignments and responses in form of job crafting and identity work (see Fig. 1).

More specifically, this article builds on the analysis of government workers’ (*N* = 81) stories to answer the following research questions:

1. How can digitalization support professional development by inducing different experiences of and responses to work-identity (mis)alignments?
2. How can digitalization hinder professional development by inducing different experiences of and responses to work-identity (mis)alignments?

## Methods

### The Method of Empathy-Based Stories

The qualitative data in this study were collected by using the method of empathy-based stories (MEBS) (e.g., Särkelä & Suoranta, 2020; Wallin et al., 2019). In the MEBS, the participants write short imaginary texts based on an introductory script (i.e., a frame story) designed by the researcher. There are always at least two different versions of a frame story, which differ with respect to one element (e.g., time frame, how the described situation proceeds, or from whose perspective the story is written). Because of this variation, the researcher can examine how the stories change when one element is altered. Thus, like other similar story-completion methods (e.g., Clarke et al., 2019), the MEBS differs from traditional qualitative self-report methods, such as interviews, because the participants are instructed to write about hypothetical scenarios instead of writing about their own experiences.

Although the MEBS can be characterized as a novel data collection method (especially in international contexts), it has a long interdisciplinary history which traces back to the 1970s and the discussion concerning the use of deception in experimental studies. This discussion prompted researchers to develop alternative methods, which preserves the ‘logic’ of experimental study design while mitigating some ethical issues (e.g., Eskola, 1998; Ginsburg, 1978; Mixon, 1972). Specifically, the experimental ‘logic’ is preserved by using one variating element between different frame story versions, allowing the researcher to study how the variation influences the produced stories, while at the same time giving the participants the freedom to use their thinking to consider different options and decide how to respond to the researchers’ prompts. Since the 1980’s the MEBS has been used and developed especially in Finland, where it has established its place among qualitative research methods (e.g., Eskola & Wallin, 2015; Särkelä & Suoranta, 2020; Wallin et al., 2015, 2019).

As the participants in MEBS research are instructed to imagine themselves in some prescribed, imaginary situation and/or role, empathy-based stories do not necessarily describe the participants personal, “real” (lived) experiences (for instance, their own emotions and actions), but instead seek to illustrate how the participants make sense, understand, or conceptualize a phenomenon. The imaginary nature of the stories entails that the narrative approach adopted in this study differs from more traditional narrative research (and especially from narrative inquiry), where the focus is on studying participants’ lived experiences or life histories, which can also be labeled as ‘Big Stories’ (e.g., Bamberg, 2006; Clandinin & Rosiek, 2012). Furthermore, although empathy-based stories usually are rich in their meanings and have a plot with a story sequence (see e.g., Elliott, 2012), they are also often relatively short, less descriptive, and more straight-forward compared to longer narratives produced for instance during narrative interviews. Thereby, adopting a narrative approach in MEBS research makes it necessary to take a broad view regarding what accounts as a story, sharing many similarities with the notion of “small stories” (e.g., Bamberg & Georgakopoulou, 2008), i.e., that a story can be hypothetical, future-oriented, and short.

Nevertheless, MEBS research shares the view with narrative research that stories, long or short and “real” or “not-real”, have the ability to produce meaning, illustrate individual sense-making and even influence how we understand ourselves and others (e.g., Bruner, 2004; Lieblich et al., 2011). The stories are not seen as merely fictional – they are seen as based on culturally and socially shared genres and meanings. In other words, the context sets the scene for what is possible to imagine (e.g., Bruner, 2004; Spector-Mersel, 2010).

Moreover, imagination and storytelling in MEBS research is deeply related to “possibility thinking” (Given, 2012), enabling individuals to imagine possible selves, futures, and connections—or, as Särkelä and Suoranta (2020, 410) stated, empathy-based stories “can be seen as “an array of “real utopias” which are not-yet realized alternatives of the state of events, but which are nevertheless achievable”. Therefore, empathy-based stories should not be taken as descriptions of “how something is” but rather as constructions of “how something might be” or “how something might have been”, thereby advancing our sense of possibilities (e.g., Meretoja, 2017) and portraying the participants’ preconceived notions and shared cultural beliefs (Wallin et al., 2019).

Building on these ideas, the MEBS was well suited for our purposes because it allowed the participants to use storytelling and their imagination when sharing their perceptions, enabling us to recognize novel perspectives, illustrate variations in the participants’ understandings, and explore possibilities and future visions that might not surface with other methods (e.g., Wallin et al., 2019). Moreover, using the MEBS also gave the participants an opportunity to distance themselves from the subject and express themselves with less external pressure than personal interviews, which can be seen as assets in our study as topics concerning digitalization, professional development, and identity are value-laden and possibly sensitive and emotive. Adopting a self-distanced perspective has been shown to, for instance, help people to reframe negative experiences, reconstrue their experiences in ways that reduce distress and reduce emotional reactivity when reflecting future negative events (e.g., Kross & Ayduk, 2011; White et al., 2019). In addition to self-distancing, in MEBS research there is usually minimal interaction between the researcher and the participants, which may help inhibit the participants from producing only socially desirable answers.

### Participants

The data were collected from 81 Finnish government workers in the spring of 2017. Participants were recruited by emailing the attendees (*N* = 790) of a national conference organized for government workers. The researchers also directly contacted some randomly selected government organizations. The participants’ ages ranged from 18 to 67 years (*M* = 50.1, *SD* = 9.48), with 65% born in the 1950s and 1960s. Thus, considering the age of the participants, our sample is close to the mean age (46,3 years) of all government workers in Finland (in year 2017). Of the 81 participants, 59.3% were female and 39.5% were male; one participant did not disclose their gender. Considering the gender of the participants, our sample includes more female compared to all government workers in Finland (female 49%, male 51%). The participants’ work positions ranged from assistants to managers, with executives and managers as the most common positions, followed by specialists and inspectors. Of the participants, 11.1% carried out administrative tasks, 37.0% were experts, 21.0% were senior experts, and 27.2% held managerial positions. Three participants (3.7%) did not report their work title. Similar to our sample, senior experts (34,9%) and experts (34,2%) represent the most common work positions of all Finnish government workers in 2017, however our sample over-emphasizes managerial positions, which represent only 2,45% of all government workers.

We focused on government workers because administrative work is highly influenced by digitalization (Frey & Osborne, 2017). Government workers are constantly dealing with the trend of digital modernization in the public sector, referred to as electronic government or e-government (e.g., Reece, 2008), both in Finland (e.g., Ministry of Finance, 2020) and worldwide. According to the Digital Economy and Society Index (European Commission, 2020), Finland is a digital leader in the European Union and is among the top ten countries in Europe in the digital delivery of public services (eGovernment Benchmark, 2020). Thus, Finnish government workers represent a suitable employee group for this study.

### Data Collection

We used two frame stories where we asked participants to imagine themselves in the future and in a situation in which digitalization had either supported or hindered the professional development of an imaginary person named Charlie:

Positive frame story:

Imagine that the year is 2025. Charlie is thinking about his/her career and notices that digitalization has supported his/her professional development. Imagine yourself in his/her situation and describe why s/he believes that digitalization has had a positive influence on his/her professional development.

Negative frame story:

Imagine that the year is 2025. Charlie is thinking about his/her career and notices that digitalization has hindered his/her professional development. Imagine yourself in his/her situation and describe why s/he believes that digitalization has had a negative influence on his/her professional development.

The topic of the frame story is likely to be familiar to participants: Finnish government workers encounter information on digitalization and professional development both at work and in public conversations. Thus, the design of the frame stories was guided by the idea that the frame stories would be as simple and short as possible to facilitate participants’ imagination. Moreover, given that our purpose was to explore the participants’ underlying perceptions and assumptions, too much detail and direction could have limited the variation and richness of the data (see e.g., Braun et al., 2019). Thereby, the participants could freely use their own interpretations when writing the stories and write the stories from any perspective (e.g., profession, career phase). Charlie (“Kaino” in the original Finnish versions) was chosen as the protagonist’s name because it is a gender-neutral name. Moreover, the Finnish language does not have gender-specific personal pronouns; the same personal pronoun (*hän*) is used to refer to all genders. Therefore, the participants could leave Charlie’s gender unspecified in the stories. Gender neutrality was chosen because it was not within our interest to explore gender-related meanings. However, in this article, we use the generic “he” when referring to Charlie to facilitate the clarity and readability of the text. Interestingly, even though most stories (*n* = 70 out of 101 stories, 50 positive and 20 negative stories) were written from Charlie's perspective or from a passive stance (*n* = 11, 8 positive and 3 negative stories), some stories (*n* = 20, 10 positive stories and 10 negative stories) were written from the participants’ own perspectives, thereby reflecting the stories’ subjectiveness.

The data collection started with a pilot study that tested the two frame stories. In this pilot study, ten government workers provided handwritten stories for either the positive or negative version of the frame story during a face-to-face meeting at their workplace. We decided to include the pilot data in the final data set because the analysis of these pilot stories showed that the frame stories worked well; the stories answered the research questions and were written according to the assignment. After the pilot phase, the data were collected face to face in one organization (18 participants) and with an e-form that was distributed to the participants via email (53 participants). Thus, overall, the final data (81 voluntary participants) consisted of stories written during a face-to-face situation (28 participants, including ten stories from the pilot study) and stories submitted through e-forms (53 participants). The participants could choose to write their stories according to the positive or negative frame story or both (excluding the pilot study, in which the participants were randomly given one frame story version). They were also requested to report their year of birth, gender, and work position. They were given an unlimited amount of time to write their stories and could continue writing later if needed.

### Data Analysis

The participants (*N* = 81) wrote a total of 101 stories. The majority of participants (59.3%) wrote a positive story, 16.0% wrote a negative story, and 24.7% wrote both types of stories. The total word count of the stories was 15,202, and the length of the stories ranged from 13 to 870 words. On average the length of the positive stories was 155 words (*M* = 122.5, *SD* = 132.25), and the negative stories 142 words (*M* = 120, *SD* = 90.05). The longest positive story was 870 words and the shortest 17 words, and the longest negative story was 361 words and the shortest 13 words.^2^ The data were transcribed and NVivo version 11 was used in the coding of the data.

We analyzed the data through *analysis of narratives* aiming at locating “common themes or conceptual manifestations among the stories collected as data” (Polkinghorne, 1995, p. 13; see also Bruner, 1985). Data analysis had two main stages. In the first stage of analysis, we used thematic analysis (Braun & Clarke, 2006), and in the second stage of analysis, we constructed typifications based on our thematic analysis. All the six phases of thematic analysis (Braun & Clarke, 2006) were present in our thematic analysis, however, as Braun and Clarke (2006) notes, thematic analysis is not a linear but a recursive process, involving a back-and-forth movement between the different phases. This applied to our analysis, which involved several rounds of analysis and used a combination of both inductive (data-driven) and deductive (theoretical) thematic analysis.

We began the thematic analysis by reading the stories multiple times to obtain an overall sense of the data. We first sorted the participants’ stories according to the frame story version such that participants’ positive and negative stories were analyzed separately. All the stories were written according to the assignment, i.e., the positive stories only yielded ‘positive’ stories, describing how digitalization supported professional development, whereas the negative stories only yielded ‘negative’ stories, describing how digitalization hindered professional development. At this first step of thematic analysis, we used an inductive approach, i.e., we identified initial codes, sub-themes, and themes in relation to how digitalization was described as inducing work-identity (mis)alignments. Thus, we identified themes related to how digitalization was described as changing Charlie’s work (work tasks and work practices), how he positioned himself to the changes (approving, critical, inconsistent) and how Charlie was described as a professional (personality, interests, needs, values, competencies). Themes were formed from text segments comprising one or more sentences by sorting, collating, and reviewing codes and sub-themes.

As we proceeded with the analysis, our understanding of the phenomenon deepened in a classic ‘hermeneutic circle’ (e.g., Gadamer, 1975), and our analysis gained a more deductive nature; we connected the ways in which Charlie responded to the (mis)alignments to the constructs in the theories on job crafting and identity work. Thus, at this second step of thematic analysis, we identified how the stories illustrated different ways Charlie engaged in job crafting (approach/avoidance task, relational and cognitive crafting) and identity work (maintaining, transforming). During the whole analysis process (inductive and deductive) the identified themes were continuously checked against each other, and the original data set to ensure that they are coherent, and that the analysis and the data match each other.

To illustrate the variation found between the stories and the storied nature of our data (in line with the idea of analysis of narratives, Polkinghorne, 1995), during the second stage of analysis, we constructed typifications based on our thematic analysis. Whereas in the thematic analysis the aim is to identify relevant themes and patterns, a typification or type is a result of comparing and contrasting cases, with the aim to differentiate cases that are similar to each other (e.g., Kuckartz, 2014). As the idea of variation is important in MEBS research (i.e., how changing one central element in the frame stories influences the participants’ stories), the basis of our typification is derived from the frame story versions (positive/negative) as well as thematic analysis. In other words, the stories were typified according to similarities and differences found between the stories regarding the initial experiences of work-identity (mis)alignments, Charlies’ responses and whether he was described as developing professionally.

In this stage two of the analysis, we identified four typifications. The positive stories were divided into two typifications: the thriving developer (*n* = 46) and the loyal transformer (*n* = 17), and the negative stories were divided into two typifications: the stagnant self-doubter (*n* = 25) and the career crafter (*n* = 4) (see Table 1). Nearly all the stories could be included in these typifications, except for nine stories which were excluded as they were too short and/or did not contain necessary information. It is also noteworthy, that the typifications are constructions, and thereby all the stories ascribed to a certain typification may not relate to the typification in the same way (e.g., describe all the (mis)alignments or responses that are illustrated). Nevertheless, they all share some basic characteristics: in the thriving developer typification all the stories describe only positive experiences (work-identity alignments), whereas in the loyal transformer typification the stories also described work-identity misalignments induced by digitalization. However, both typifications describe how digitalization was seen as supporting professional development. The stagnant self-doubter and the career crafter both share the view that digitalization hindered professional development by inducing work-identity misalignments, however the career crafter typification differs from the stagnant self-doubter typification in that despite of the negative experiences Charlie eventually managed to experience work-identity alignment and develop professionally.

**Table 1:** Summary of the typification’s main characteristics

	*POSITIVE FRAME STORY:* *Digitalization supporting professional development*		*NEGATIVE FRAME STORY:* *digitalization hindering professional development*	
	***The thriving developer***	***The loyal transformer***	***The stagnant self-doubter***	***The career crafter***
**Initial experience**	Work-identity alignment	Work-identity misalignment	Work-identity misalignment	Work-identity misalignment
**Response**	Maintaining identity work	Transformative identity work	Maintaining identity work	Transformative identity work
	Approach crafting	Approach crafting	Avoidance crafting	Career crafting
**Storyline**	PROFESSIONAL DEVELOPMENT	PROFESSIONAL DEVELOPMENT	PROFESSIONAL STAGNATION	CAREER TRANSITION & PROFESSIONAL DEVELOPMENT

## Findings

We present the findings in two sections according to the positive or negative frame story versions. In each section, two typifications demonstrate how Charlie experienced and responded to work-identity (mis)alignments by engaging in identity work and job crafting. Thus, the two first typifications (thriving developer and loyal transformer) exemplify stories written as a response to the positive frame story (digitalization supporting professional development), and the other two (stagnant self-doubter and career crafter) exemplify stories written according to the negative frame story (digitalization hindering professional development). The main characteristics of each typification are summarized below in Table 1.

### Digitalization Supporting Professional Development

*The thriving developer.* In participants’ most positive stories, digitalization supported Charlie’s professional development by transforming work tasks and practices to better match Charlie’s career aspirations, interests, competencies, and values. As such, participants described digitalization strengthening Charlie’s professional identity; it aligned with his personal preferences, offered opportunities to fulfill his interests, and fostered a sense of authenticity and self-continuity. Thus, participants described Charlie as a thriving developer who enjoyed the possibilities brought by digitalization regarding his work and professional development. As digitalization was described in these stories as inducing experiences of work-identity alignment, professional development was mainly related to how Charlie engaged in maintaining identity work and approach crafting to strengthen the aspects of work and self that he experienced as meaningful.

In particular, participants’ stories emphasized how digitalization offered Charlie opportunities to use his core competencies, focus on the most important aspects of his work and realize his full potential. The stories illustrated how the automation of routine work tasks (such as reporting and information sharing) and the integration of various information systems resulted in increased work quality, better information flow, and higher efficiency. All this made it possible for Charlie to focus on relevant, interesting, and creative work tasks allowing him to use and develop his competencies: “Almost all my work tasks are demanding or very demanding. Now I really feel like my work has a purpose, and all the pencil pushing and pointless emailing is left behind” (P48).

Participants envisioned Charlie working with various applications of AI, robotics, and solution databases and connected it to changes for the better in his work. These digital technologies would take over the “dull routine work”, while liberating Charlie to focus on cognitively demanding tasks, such as in making decisions and in providing solutions and analyzes: “At first software robots only did basic data verification, but by 2025 they are already capable of statistical analysis. These work tasks were replaced by other, more meaningful work tasks (which were still unautomatable).” (P38).

Charlie was framed as a proactive and self-directed worker who believed in his capabilities and was optimistic and eager to learn and develop. Although digitalization required him to constantly learn and develop, this was not perceived as a burden but a possibility and opportunity that increased his motivation: “Digitalization has also brought completely new opportunities for the development of work and brought completely new work tasks that motivate Charlie to strive forward” (P30).

Participants described how digitalization entailed adoption of technical tools and devices allowing remote work, and that Charlie enjoyed and valued the autonomy and flexibility in choosing when and where to work. He also took full advantage of digitalization to further his skills and career. For instance, he was described as actively attending courses and digital workshops, watching work-related videos, and reading blogs. Thus, in the thriving developer typification, Charlie’s engagement with digitalization was described to improve the content of work tasks, broaden his skills and competencies, and render his professional role more important and meaningful —that is, digitalization provided him with various task-crafting opportunities that supported his professional development.

In addition to increasing opportunities for task crafting, digitalization was described as creating possibilities for relational crafting*.* This was shown, for instance, in how Charlie was often described as a forerunner who helped his colleagues with problems they faced with digitalization. Thus, being interested in technology and among the first to apply digitalization to his work helped Charlie build relationships with his colleagues and gain confidence and appreciation. Similarly, digitalization also provided opportunities for Charlie to ask colleagues for feedback and advice, grow his professional networks, solve problems together with colleagues regardless of time and place, and find and share work-related knowledge: “Web conversations with colleagues around the world are particularly rewarding. Sometimes, even several times a week, we discuss work-related themes and share tips on how to act in different situations” (P80).

*The loyal transformer.* Although participants’ positive stories mostly emphasized how digitalization strengthened Charlie’s professional identity, some stories illustrated how Charlie also experienced misalignments between his professional identity as digitalization conflicted with his competencies, values, or interests. For instance, the stories expressed concerns about how digitalization might dehumanize work as robots replace human labor. Additionally, the stories illustrated how Charlie felt that digitalization disturbed his work as he had to deal with various challenges, such as malfunctioning software, demanding work tasks, and insufficient (digital) skills: “It would be good to be able to focus on preparation that required expertise instead of pondering how things should be done on a computer” (P28). Digitalization was described to require a new ‘technical’ way of thinking, and adopting such technical thinking was considered not to be easy. Also, Charlie could lack skills to manage information flows and the new ways of online collaboration: “Working on shared documents is still hard for Charlie and in his opinion, there should be someone who would be in charge of the document” (P45).

Despite the contradictions between digitalization and his professional identity, these stories emphasized that Charlie considered digitalization and related professional development as necessary to stay professionally current. Like in the “thriving developer” typification, Charlie was described as a growth-minded and proactive worker, who was committed to his organization and profession. Although Charlie possessed some critical thoughts regarding digitalization at work, he considered it vital to “stay on top of development by doing whatever is needed” (P43). Thus, Charlie was described as a loyal transformer, who engaged in approach cognitive crafting and/or transformative identity work to reframe his perceptions of his work and identity: “With digitalization, he has had to reflect on his own starting points and ways of doing work, which has brought a different perspective to work” (P19). In these stories, cognitive crafting was related to how Charlie reframed his work role, envisioned the challenges positively, and emphasized the positive qualities of work to realign his professional identity with the transformed work context. He also gained positive and encouraging experiences from engaging with digitalization (which enhanced his self-confidence and self-efficacy), received social support, and eventually learned how to best exploit digitalization in his work. Thus, as it was important for Charlie to remain a skilled and competitive employee, in the end “Charlie was satisfied and relieved that he did not resist an inevitable development, which would have slowed down his professional development and weakened the possibilities to succeed in his work in the year 2025” (P36).

Thus, the positive stories demonstrated how digitalization supported Charlie’s professional development by resulting in a strengthened or redefined professional identity and offered him possibilities to engage in approach job crafting. Consequently, in these stories digitalization was related to feelings of competence, meaningfulness, satisfaction, and thriving at work.

### Digitalization Hindering Professional Development

*The stagnant self-doubter.* Similar to the misalignments described earlier in the “loyal transformer” typification, the negative stories shared the view that digitalization and the changes it brought contradicted the way Charlie defined himself as a professional. However, in contrast to the positive stories, where Charlie eventually managed to reach a work-identity alignment and were described to develop professionally, the majority of negative stories described Charlie as a stagnant self-doubter, unable to resolve the misalignment between his professional identity and work context and thus, develop professionally.

The negative stories emphasized how digitalization transformed Charlie’s work in a way that did not correspond with his professional interests, competencies, and values, and thus led to experiences of work-identity misalignment. For instance, digitalization was described as diminishing the aspects that he valued in his work, such as the social nature of work, replacing them with aspects that he did not value or was not competent in, such as dealing with technical problems:

Communication with the closest colleagues is no longer as close as before; we rarely meet face to face, only once or twice a week, and many things need to be handled electronically or in virtual meetings. (N32)

Charlie would like to do his “basic tasks” well, but it has not been possible for a long time without first having to learn all the “nerd stuff.” Work has become burdensome and repulsive. (N8)

Charlie felt that digitalization merely disrupted his work, decreasing the possibilities to use his full potential and fulfill his professional interests. On one hand, he was not able to focus on his core work tasks; constantly learning new, unfinished, and often malfunctioning systems took all his work hours. Such learning hindered Charlie’s professional development as it did not allow Charlie to advance in a meaningful mastery of his work or in a more enabling definition of himself as a professional. On the other hand, the stories also described how the automation of (routine) work tasks resulted in him not being able to manage his remaining, more demanding, and complex information analysis and decision-making tasks properly. He was supposed to do things differently from what he was used to and felt incompetent to tackle cognitively demanding tasks: “Routines are handled in the background by systems and software robots. Of course, it is still necessary for me to draw conclusions and evaluate the results and decisions. I am required to do very different things than before and therefore I am not able to properly handle my work tasks, which is not motivating.” (N32).

Charlie was described as old-fashioned and uninterested in technology, and the stories also often described how Charlie lacked personal resources needed to develop professionally, such as self-directedness and proactivity. He was satisfied with his old work routines and wanted to stay in his comfort zone rather than challenge himself:

“Oh, it was much better before,” Charlie thought while opening the door to his workplace. “I knew precisely in advance what my day would include in terms of work tasks, and my work was scheduled. I got to focus on reports and produce them so that they could be analyzed by others. … We have always done things a certain way, and things have worked well. I feel I work best when I get to do things that I am familiar with, and that is why I was hired here.” (N22)

The negative stories also frequently illustrated how the participants associated digitalization with problems in organizational implementation, such as lack of sufficient organizational support and resources. Charlie felt like he received insufficient training and social support to develop professionally, and digitalization was often associated with malfunctioning and complicated technologies, continuous interruptions, and increased workload. Hence, in addition to digitalization itself, its poor implementation further impacted negatively on Charlie’s professional development: “When digitalization first began, the workload increased significantly, guidance was deficient, and systems were introduced incomplete and inoperable” (N65).

Digitalization led to feelings of incompetence, frustration, and constant worry over not succeeding in the job. The stories frequently described how Charlie felt useless, redundant, and undervalued: “With digitalization, Charlie’s previously valued solutions have become unnecessary, and Charlie’s expertise is hardly needed anymore” (N25). In five of the 33 negative stories, digitalization and automatization led also to unemployment:

One after another, the office staff received a final account. Those lucky ones who were in their 60s at the start of the changes were allowed to retire. We in our 50s, on the other hand, were left with nothing, with working life remaining well over 10 years, but age itself was a barrier to getting a job. I have already been unemployed for several years. I cannot see that the situation is going to change. (N65)

Although Charlie expressed reluctance and critical thoughts regarding digitalization, only one story described how Charlie consciously resisted the changes by engaging in avoidance task crafting. In this story, digitalization had replaced face-to-face customer service with digital forms, and because Charlie was not satisfied with the changes, he decided to drag his feet during the face-to-face meetings. Thus, he failed to meet his profit target and was laid off.

Most negative stories described the frustrating situation and adopted a deterministic view of technology: Charlie felt like digitalization was an inevitable fact of life. He felt like he had no control over the situation and could only passively adapt to it: “At some point, someone pressed delete, and everything was gone. Someone reversed the logic of the system, and digitalization controls life even though digitalization should support life” (N40).

Thus, the majority of negative stories illustrated how Charlie was reluctant or unable to realign his work and identity. Charlie felt obliged and forced to use technology in his work, and he struggled to cope with the frustrating situation by engaging in avoidance cognitive crafting by distancing or withdrawing himself from digitalization: “At home, Charlie still doesn’t want to use a computer even though his wife has bought one” (N64).

*The career crafter.* Although in most negative stories, Charlie did not manage to resolve the work-identity misalignment and was left in a liminal state, four stories framed a more optimistic outcome. In these stories, Charlie distanced himself from digitalization and his present job and, by finding a new career path, managed to realign his work and identity by engaging in non-digital and creative work in which he felt competent: “Fortunately, Charlie feels that he is an important person in the world of small children because he has time for them” (N37). Thus, even if digitalization hindered Charlie’s professional development in his original job, it encouraged Charlie to move towards a career and a professional identity that better fits his life goals and values. On this new career path, professional development might become possible again.

## Discussion

In this study, we have illustrated government workers’ perceptions regarding the relationship between digitalization and professional development. Based on empathy-based stories, we introduce four typifications to demonstrate the various ways by which the participants perceived that digitalization could support or hinder professional development by inducing different experiences of and responses to work-identity (mis)alignments. In sum, *the thriving developer* typification shows how digitalization may induce experiences of work-identity alignments, allowing the worker to engage in maintaining identity work and approach job crafting, and thus supporting professional development. Similarly, *the loyal transformer* typification describes how digitalization can support professional development, however, in this typification the process is not as straight-forward as in the thriving developer, as the worker may also experience some work-identity misalignments and thus, is required to engage in transformative identity work and job crafting to realign his identity and transformed work context. Similarly, both *the stagnant self-doubter* and *the career crafter* typifications illustrate how digitalization may induce work-identity misalignments, however the stagnant self-doubter is a typification of professional stagnation as the worker is not able to realign his identity and work context, whereas in the career crafter the worker eventually manages to find a work-identity alignment and thus, develop professionally.

To the best of our knowledge, this is the first study to examine the relationship between digitalization and professional development using an identity-centered approach. Also, this study uses a novel data collection method, the MEBS, which takes advantage of the use of imagination in storytelling, thereby enabling us to recognize new perspectives and participants’ possible ways of thinking. Thus, this study contributes to existing knowledge and theories in several ways, provides some practical implications and opens future research avenues.

### Theoretical Contributions

First, this study extends existing research on professional learning and development by illustrating how digitalization and workers’ identities influence it. The findings show how digitalization can influence workers’ experiences of work-identity (mis)alignments by affecting how they define themselves at work and their possibilities to work in a meaningful way. Thus, the findings illustrate how individuals differ in what they believe to constitute meaningful work that aligns with and validates their professional identities, and thereby highlights the importance of individual subjectivities in understanding professional learning and development (e.g., Billett, 2010). For instance, in the positive stories, Charlie viewed replacing routine work with more challenging work tasks as something desirable, whereas in the negative stories, this was perceived as a threat to his professional identity. These findings resonate with the findings of earlier studies that illustrate how digitalization can both reinforce and threaten workers’ identities (e.g., Mishra et al., 2012; Nach, 2015; Stein et al., 2013). For instance, a recent study (Långstedt, 2021) illustrates how automation and the implementation of intelligent technologies (such as AI) at work may lead to a work-values misalignment, as the pre-automated, more routine, and structured work relates to different values, needs and skills than the new work environment after automation, characterized by more creative and investigative work. Thereby, our research builds an interdisciplinary bridge between studies that have explored how technologies and work interact with one’s identity, and studies emphasizing the role of subjectivities and professional identity in professional development, suggesting that it is important to understand how digitalization influences work-identity interactions to best support workers’ professional development.

In addition to illustrating the importance of work-identity interactions in developing professionally, the findings illustrate how professional development is a subjective construction and can be understood in various ways. In particular, the different understandings regarding what accounts as professional development was apparent when comparing the positive and the negative stories. In both the positive and the negative stories, professional development was related to everyday work practices, however in the positive stories also engaging with digitalization (e.g., adoption of new software, learning new digital ways of working) was seen as a part of Charlies’ professional development, whereas in the negative stories engaging with digitalization was considered as merely a burden and hindering his professional agency and development. Thus, these different conceptualizations highlight the importance of considering the subjective evaluations and positions of workers when striving to support their professional development.

Second, scholars have extensively studied how workers strive to maintain a coherent and positive identity and how they respond to work-identity misalignments (e.g., Alvesson, 2010; Caza et al., 2018; Fuller & Unwin, 2017; Pratt et al., 2006; Sveningsson & Alvesson, 2003). This study contributes to this body of research by illustrating, through three typifications (the loyal transformer, the stagnant self-doubter, and the career crafter), differences in how workers might respond to digitalization and the misalignments it might induce between work and identities. Moreover, as previous studies on work and identities have focused more on work-identity misalignments than alignments (e.g., Kira & Balkin, 2014), this study provides insights into how workers might experience and respond to work-identity alignments, as demonstrated by the “thriving developer” typification.

In particular, the findings contribute to previous theories on identity work and job crafting (e.g., Bruning & Campion, 2018; Kira & Balkin, 2014; Lazazzara et al., 2020) by illustrating how digitalization could provoke workers to engage in identity work and how digitalization could induce both approach and avoidance crafting. For instance, the “thriving developer” typification shows how digitalization might lead workers to engage in maintaining identity work and in approach task and relational crafting. By contrast, “the stagnant self-doubter” typification illustrates how digitalization might lead workers to merely cope with the situation and to engage in avoidance job crafting, such as distancing and withdrawal. These negative stories resembled Alvesson’s (2010, p. 200) “self-doubter image,” as Charlie remained “riddled by the unpleasant and pervasive experiences of insecurity and anxiety.” Thus, the “stagnant self-doubter” typification illustrates how identity work and job crafting may not always culminate in positive identity states (e.g., Caza et al., 2018) but may also result in subjugated identities (Kira & Balkin, 2014), “anti-identities” (Nach, 2015), and liminal or “in between” professional selves (Beech, 2011), thus hindering professional development. However, similar to the “loyal transformer” typification, in the “career crafter” typification, transformative identity work was seen as a struggle with an element of mild heroism (Alvesson, 2010), as Charlie eventually managed to develop professionally and construct a positive professional identity despite the frustrations and contradictions caused by digitalization. These findings demonstrate how workers can resolve the work-identity misalignments created by digitalization by engaging in career crafting, referring to “an individual’s proactive behaviors aimed at optimizing career outcomes through improving person-career fit” (De Vos et al., 2019, p. 129). Likewise, the “career crafter” typification also illustrates how workers compare their present and alternative commitments when their current commitments are no longer satisfactory, as illustrated in the “reconsideration of commitment” dimension of the identity status models (e.g., Crocetti et al., 2008; Mancini et al., 2015).

The various ways by which workers may respond to work-identity (mis)alignments induced by digitalization highlights the importance of recognizing workers’ professional agency in professional development, i.e., acknowledging how workers are active agents who evaluate changes and decide how to involve and position themselves within said changes (e.g., Vähäsantanen & Billett, 2008; Vähäsantanen & Eteläpelto, 2011; Ylijoki & Ursin, 2013). Although most earlier studies have focused on teacher profession and educational reform as their context, their findings resonate with the findings of this study, showing how workers can manifest their agency in multiple ways in changing work contexts. For instance, Vähäsantanen and Billett (2008) show how vocational teachers may adopt five different personal strategies (professional development strategy, passive accommodation strategy, active participation strategy, balancing act strategy, and withdrawal strategy) when negotiating their professional identities in a reform context. Along similar lines, Vähäsantanen and Eteläpelto (2011) demonstrate how teachers may during educational reforms exercise their professional agency by maintaining or transforming their professional identities. Thus, although reforms, such as digitalization, are often planned and organized from the top-down, the workers’ have still various ways by which they can exercise their agency amid changes.

These various ways workers can exercise their agency raises a question regarding what is needed in ‘managing’ professional development successfully amid (digital) changes. The stories of this research especially shared a view that workers’ need to proactively engage with digitalization and to actively take initiative to develop one’s skills and competencies to stay professionally current, ensure employability and to avoid falling in the wayside professionally. In this sense, the findings align with previous studies suggesting that the “ideal ‘new’ employee is a self-directed, proactive, networking entrepreneur, taking responsibility for his or her own performance and development” (van den Heuvel et al., 2010, p. 124; see also Dachner et al., 2021) and that the introduction of new technologies requires “new” skills of employees, such as adjustment to new (digital) work practices (e.g., Vallo Hult & Byström, 2021). Thus, the stories replicated the culturally shared view and the dominant intervention strategy focusing on building employees’ education and skill levels and fostering their adaptivity so that they can cope with new technologies and remain employed (Parker & Grote, 2020).

Indeed, the need to constantly engage in self-initiated or self-directed learning in today’s digital working has been recognized in several studies on workplace learning (e.g., Lemmetty & Collin, 2020), and proactivity is also suggested to be incorporated in the definition of employee development (Dachner et al., 2021). However, on the other hand, the negative stories in this study also illustrate the ‘dark side’ of proactivity and self-directed learning, i.e., what may happen if workers lack qualifications associated with ‘the ideal new employee’, and if too much responsibility for learning is thrusted on workers. The stagnant self-doubter typification illustrated how every worker may not have the capabilities or willingness to proactively develop themselves amidst changes, and how sometimes their opportunities can be restricted by the organization. Thereby the findings align with the findings of previous studies, demonstrating how self-directed learning is not always seen as something positive, but may instead be regarded as a burden and a stressful obligation (e.g., Lemmetty & Collin, 2020). More broadly, the ‘self-doubter’ image of identity in this typification illustrates how “a constant pressure on individuals to adapt and be responsive means that the social preconditions for building character and identity are not there anymore” (Alvesson, 2010, p. 200), and consequently workers may find it difficult to find meaning in their work. This implies that emphasizing workers’ proactivity and professional agency in professional development should not mean that workers are left completely on their own, trusting that “employees themselves would somehow find their way if their work changed or jobs disappeared” (Saari et al., 2019, p. 300). If workers are seen as solely accountable for their professional development, there is a risk that learning and growth opportunities are not equally distributed and a system of exclusion evolves (Dachner et al., 2021).

Therefore, the findings of this research support earlier studies on professional learning and development, emphasizing the need for a “shared partnership approach” (e.g., Billett, 2001; Dachner et al., 2021), i.e., the acknowledgement that to best support professional development a focus needs to be on both workplace affordances and on individual’s characteristics and engagement. In the context of digitalization, this entails that a “tool view of technology” (e.g., Kim et al., 2020; Orlikowski & Iacono, 2001) is too limited—that is, there are no determined outcomes of engaging with digitalization. Rather, to understand the relationship between digitalization and professional development, adopting a “proxy view” or an “ensemble view” of technology is necessary. Both views acknowledge the importance of the human agency of technology adopters; however, the ensemble view also emphasizes “the importance of social contexts within which technological artifacts are formulated, enacted, interpreted, and appropriated” (Kim et al., 2020, p. 6).

### Practical Implications

In accordance with previous discussions (e.g., Goller & Harteis, 2017; Vähäsantanen & Billett, 2008; Vallo Hult & Byström, 2021), this study emphasizes the need for greater consideration of individual needs and human agency when seeking to support the professional development of workers in certain changing contexts, such as digitalization. As technologies are usually introduced in organizations in a top-down fashion (e.g., Hornung et al., 2010), we propose that when seeking to support workers’ professional development in a digitalized working life, organizations should stimulate a work environment that enables the workers to craft their work in a way that is important and meaningful to them and should support the use of flexible and adaptive digital structures (e.g., Parker & Grote, 2020). Considering the recent COVID-19 outbreak, this is especially important as the workforce is required to adapt to and cope with radical changes in the work and social environment, such as shifting to remote work environments (Carnevale & Hatak, 2020). Compared to before the pandemic, remote work, for instance, is no longer voluntary but mandatory. Therefore, providing a supportive environment (e.g., Wang et al., 2020) and listening to workers’ needs, values, and beliefs regarding their work is especially important for the workers to identify themselves as “thriving developers” instead of “stagnant self-doubters”. The interests of the organization should also be considered in job design; thus, adapting the principles of idiosyncratic deals (“i-deals”; Rousseau et al., 2016) in digitalization could benefit both the workers and their employers. In this way, the individual is given “the possibility to work in alignment with his/her work identity while striving towards organizational goals” (Kira et al., 2012, p. 49).

### Limitations and Future Prospects

The limitations as well as the strengths of this study relate especially to the data collection method. As the data were collected through the MEBS, the findings portray possible connections and perspectives rather than participants’ personal experiences. Thus, one possible criticism of using the MEBS (concerning also other similar story completion and role playing methods; see e.g. Clarke et al., 2019; Greenberg & Eskew, 1993), has been, that the data lacks realism and the stories are artificial, i.e. the data does not reflect or predict ‘real-life’ behavior. Although it is important to acknowledge this fact, it is also important to recognize that there does not need to exist a correspondence between people’s perceptions and actions for there to be value to data (see Greenberg & Eskew, 1993). The value of studies using the MEBS lies not in explaining or predicting the participants’ actual behaviors, but in presenting possible ways of perceiving the phenomenon and in discovering what kind of shared meanings and assumptions exist. Whether these perceptions and meanings are based on personal experiences is not relevant, although in this study the similarities found between the stories, and the fact that the stories were often written from the perspective of a knowledge/government worker and also from the participants’ own perspectives instead of Charlie’s, shows how the participants may draw on both personally and socially available resources in telling a story that makes sense (see also Clarke et al., 2019).

Given that most of the participants of this study could write the stories in a relatively short time anytime and anywhere, the MEBS enabled us to collect qualitative data from a wider sample than, for instance, with interviews. However, the written and imaginary format of the data can also bring some limitations regarding the quality of the data, shown, for instance in very short responses. Indeed, the frame stories may not spur imagination in the same way to every participant, and the participants’ writing fluency might also vary (see also Clarke et al., 2019). In this study, participants were government workers used to describe their thoughts and ideas in writing and they also took part in the study voluntarily. It was likely that participants would not struggle with writing the story. However, the short stories in the data might reflect some difficulties in imagining and/or perhaps, the workers’ busy schedule. Nevertheless, despite that some stories (*n* = 7) were very short (less than 40 words), most participants wrote ‘full’ stories. Additionally, even the shortest stories did describe some aspects (e.g., how digitalization transformed work) relevant for our research questions.

Moreover, although the sample size (*N* = 81) is high compared to most studies using the MEBS as a data collection method, the sample is non-representative and relatively small to generate any statistical generalizations. Furthermore, despite that the studies are based on a multi-organizational sample, all participants are Finnish government workers. Thus, the findings are context-dependent and as such, might not be applicable to other contexts (e.g., other professional fields or cultural contexts). The findings are also time-bound, reflecting the participants’ views in 2017. However, this is not to say that the findings completely lack the capacity for generalization. Indeed, in qualitative research, the concept of transferability is seen as more helpful in understanding generalization compared to a formal, quantitative understanding of generalization (e.g., Tracy, 2010). *Transferability* (e.g., Lincoln & Guba, 1985; Tracy, 2010) is achieved “when readers feel as though the story of the research overlaps with their own situation and they intuitively transfer the research to their own action” (Tracy, 2010, p. 845). Transferability can be increased by, for instance, writing accessibly, evocatively and invitationally, and thus creating a feeling in readers as if they have experienced the same thing in another context. The different typifications and illustrations presented in this study aim to increase the evocativeness of the findings and provide the readers with possibilities to recognize familiar “patterns'' or interpretations, thus increasing the resonance of the findings. Nevertheless, in the future it would be useful to situate studies in other contexts (e.g., professional field and cultural context) to complement the findings of the present study. Also, looking more closely at how workers’ work histories and demographic information (e.g., age, educational level) influences, for instance, how the workers respond to digital changes in their work and their valuations, is needed.

In this study, as in typical MEBS research (e.g., Wallin et al., 2019), the frame stories were divided into positive and negative to explore how the stories change when one element is varied. This gave us the opportunity to illustrate, for instance, how the descriptions concerning Charlie’s professional identity varied between the positive and negative stories. Although this study illustrates the findings through two opposing poles (positive/negative) and four typifications, in reality, the stories might blend and take different forms. Thus, the aim of this study is not to claim that workers identify with only these extreme cases but rather to illustrate possible scenarios. In future research, it would be useful to investigate and extend the findings using different qualitative methods, such as interviews, diary studies or observations. As empathy-based stories are relatively short and more straight-forward compared to stories collected through, for instance, interviews, exploring the phenomenon with other narrative methods might help to deepen the findings and relate the findings to participants’ lived experiences. Thus, future inquiries could identify more variations in how workers experience and respond to work-identity (mis)alignments created by digitalization. For instance, a deeper and more detailed look into how workers values, work histories, interests, and competencies influence what kind of work-identity misalignments and alignments digitalization could induce and their relationship to different contextual and individual factors would help to build a more profound understanding on the relationship between professional development and digitalization. Furthermore, although the purpose of this study was not to explore the role of emotions in digitalization and professional development, the findings clearly indicated that these are highly relevant. The stories frequently mentioned how digitalization evoked emotions ranging from frustration to satisfaction and thereby, in the future a closer look into what kind of emotions digitalization causes and how these are related to professional development would be valuable. This is in line with the findings of a recent meta-synthesis focused on the relationship between professional agency and emotions at work, suggesting that emotions play an important role in professional learning and development, especially when bound up with professional identity negotiations (Hökkä et al., 2017).

Moreover, an interesting observation in this study was that most participants chose to answer the positive frame story version. One might wonder, does this reflect that Finnish government workers in general position themselves toward digitalization at work in a more positive way, or maybe this reflects their optimistic and hopeful positioning toward the future as they were instructed in the frame story to imagine the year 2025? In the context of this study, these questions are not answerable but in future research avenues it would be interesting to delve into these questions more deeply.

## Conclusions

This study provides a novel perspective of professional development by illustrating the possible connections between digitalization and professional identity. The findings highlight the importance of recognizing how digitalization of work can threaten or support workers’ professional identities to build a supportive working environment where the workers feel like they are valued and able to develop in a meaningful way.

## Data Availability

Not applicable.

## Footnotes

1 Cognitive crafting and (transformative) identity work can be conceptually distinguished as cognitive crafting refers to how workers make changes to their perceptions about their job (e.g., thinking about the positive qualities of work), whereas identity work refers more broadly to identity negotiations related to how workers define themselves (as professionals). However, job crafting and identity work are closely related to each other as when workers engage in job crafting, the identities of the workers can also change accordingly (e.g., Kira & Balkin, 2014).

2 For simplicity, we call all the data “stories” although the shortest stories could be referred to more as ”responses”. Three stories were 17 words or less and these are excluded from the data when making the typifications. However, these stories are included in the thematic analysis as they did contain information regarding, for instance, how digitalization changed Charlies’ work and thus influenced professional development.
